# Prädiktoren für die Nutzungsintensität von Tagespflegen bei Menschen mit kognitiven Einschränkungen

**DOI:** 10.1007/s00391-021-01972-x

**Published:** 2021-09-29

**Authors:** Catharina Wasić, Elmar Gräßel, Katharina Luttenberger, Carolin Donath

**Affiliations:** Zentrum für Medizinische Versorgungsforschung, Klinik für Psychiatrie und Psychotherapie, Universitätsklinikum Erlangen, Friedrich-Alexander-Universität Erlangen-Nürnberg (FAU), Schwabachanlage 6, 91054 Erlangen, Deutschland

**Keywords:** Modell der Versorgungsinanspruchnahme, Demenz, MCI, Pflegende Angehörige, Regression, Healthcare utilization model, Dementia, Cognitive dysfunction, Family caregivers, Regression

## Abstract

**Hintergrund:**

Tagespflegen sind etablierte Versorgungsangebote für Personen mit kognitiven Beeinträchtigungen. Die Nutzungsintensität ist mit durchschnittlich 3 h/Woche jedoch gering. Bisherige Studien konnten Prädiktoren für eine Nutzung/Nichtnutzung aufzeigen. Es ist jedoch bisher unklar, inwieweit diese Faktoren auch mit der Nutzungsintensität zusammenhängen.

**Ziel der Arbeit:**

Identifikation von Prädiktoren für die Intensität der Tagespflegenutzung bei Menschen mit kognitiven Beeinträchtigungen, basierend auf dem Modell der Versorgungsinanspruchnahme nach Andersen.

**Material und Methoden:**

Grundlage sind Daten der Studie Demenz in der Tagespflege bei psychosozialer MAKS-Intervention (DeTaMAKS). Prädiktoren für die Nutzungsintensität der Tagespflege wurden mit logistischer Regression analysiert.

**Ergebnisse:**

Eine signifikant höhere Intensität der Tagespflegenutzung lag vor bei: alleinlebenden Tagespflegegästen, höher gebildeten pflegenden Angehörigen, ab Pflegestufe 2, bei längerer bisheriger Nutzungsdauer der Tagespflege, bei vermehrten psychischen und Verhaltenssymptomen des Tagespflegegasts. Die Sensitivitätsanalyse zeigte bei zusammenlebenden Dyaden zusätzlich eine höhere Nutzungsintensität bei jüngeren pflegenden Angehörigen und kürzerer Entfernung zwischen Wohnort und Tagespflege, jedoch nicht in Bezug auf den Bildungsstand des pflegenden Angehörigen und die psychischen und Verhaltenssymptome des Tagespflegegasts.

**Diskussion:**

Die Ergebnisse zeigen bestehenden Bedarf an Tagespflegeeinrichtungen, der sich durch Berufstätigkeit und das Leben ohne Partner erhöht. Neben einer guten Erreichbarkeit der Tagespflege könnten auch flexible Angebote die Intensität der Nutzung erhöhen.

**Zusatzmaterial online:**

Zusätzliche Tabellen sind in der Online-Version dieses Artikels (10.1007/s00391-021-01972-x) enthalten.

## Abstract (en)

**Background:**

Adult day care is an established concept in Germany for people with cognitive impairment; however, only a small fraction of people in need for care actually use adult day care. Studies so far highlighted some predictors for the use of adult day care; however, it remains unclear which factors are associated with the intensity of use.

**Objective:**

To identify relevant predictors for the intensity of use of adult day care using the Andersen healthcare utilization model.

**Material and methods:**

Data used were obtained within the project dementia in day care with psychosocial MAKS interventions (DeTaMAKS), which studied adult day care users with cognitive impairments and their family caregivers. A logistic regression was performed to predict frequent or low use of adult day care.

**Results:**

The following factors were significantly associated with higher intensity of use: civil status of adult care user being widowed or single, higher educational level of caregiver, higher care level, longer duration of adult day care use and more mental and behavioral symptoms of the adult day care user. The sensitivity analysis for cohabiting dyads additionally showed a higher intensity of use with a lower age of the caregiver and shorter distance between place of residence and adult day care but not with respect to educational level of the caregiver and mental and behavioral symptoms of the user.

**Conclusion:**

The results show a need for adult day care, which increases with caregivers being employed and users living outside of permanent relationships. A short distance to the adult care center as well as flexible care options may increase the frequency of use.

Tagespflegen entlasten pflegende Angehörige stundenweise durch die Betreuung eines zu Hause lebenden Pflegebedürftigen. Nur 4 % der zu Hause lebenden Pflegebedürftigen in der Bundesrepublik Deutschland nutzen eine Tagespflegeeinrichtung [15] und dies durchschnittlich für 3 h die Woche [21]. Wovon hängt es ab, wie intensiv das Angebot der Tagespflege genutzt wird? Dieser Beitrag ermittelt Faktoren, die mit der Nutzungsintensität von Tagespflegen im Zusammenhang stehen und ordnet sie in das Verhaltensmodell der Versorgungsinanspruchnahme nach Andersen et al. [1] ein.

## Hintergrund und Fragestellung

### Tagespflegen in Deutschland

Das Konzept der Tagespflege (TP) existiert in Deutschland seit 1973 [11] und ist durch §41 SGB XI als möglicher Bestandteil von Pflegeleistungen im Gesetz verankert [5]. Die Zahlen des Statistischen Bundesamtes zeigen, dass im Jahr 2019 82.639 Tagespflegeplätze in Deutschland zur Verfügung standen und 139.192 Pflegebedürftige dieses Angebot nutzten. Bei insgesamt 3,3 Mio. zu Hause lebenden Pflegebedürftigen besuchten somit ca. 4 % davon eine TP [15].

Die TP zählt zu den bekanntesten formellen Hilfeleistungen. Laut einer Umfrage kannten 56,5 % der pflegenden Angehörigen die TP [10]. Bei einer Befragung von pflegenden Angehörigen einer Person mit Demenz war die TP die zweithäufigste genutzte Hilfeleistung [20].

Ein Review konnte zeigen, dass die Tagespflegenutzung für Personen mit Demenz eine Verbesserung des Schlafes und der Verhaltenssymptome und für ihre Angehörigen eine Verminderung der Pflegebelastung bedeuten kann [19]. Gleichzeitig war die Wahrscheinlichkeit eines Heimübertritts bei Nutzung einer TP signifikant erhöht, wenn keine anderen formellen Hilfeleistungen in Anspruch genommen wurden. Diese Ergebnisse konnten durch weitere, aktuelle Studien bestätigt werden [14, 16, 18].

### Nutzungsintensität von Tagespflegen: Rahmenkonzept

**Figure 1:**
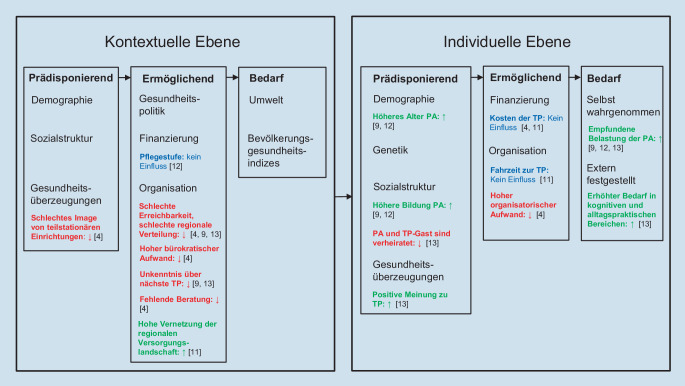

Nach dem Verhaltensmodell der Versorgungsinanspruchnahme von Andersen et al. [1] bestimmen *prädisponierende, ermöglichende *und* Bedarfsfaktoren* die Nutzung eines Angebotes im Gesundheitssystem. Die Faktoren können dabei sowohl auf kontextueller Ebene, d. h. auf Ebene der Region, als auch auf individueller Ebene betrachtet werden. *Prädisponierende* Faktoren sind bestehende Merkmale einer Person bzw. Region, die eine mögliche Nutzung/Nichtnutzung beeinflussen. Die *ermöglichenden* Faktoren stellen Merkmale dar, die den Zugang zu Versorgungsangeboten begünstigen oder beeinträchtigen. *Bedarfsfaktoren* charakterisieren den Bedarf an Versorgung, der zu einer tatsächlichen Nutzung führt. In Abb. 1 sind die bisherigen Forschungsergebnisse zur Nutzung von TP abgebildet und in Bezug auf das Andersen-Modell [1] eingeordnet.

Bisher ist jedoch unklar, inwieweit diese Faktoren auch mit der Nutzungsintensität von TP bei Menschen mit kognitiven Beeinträchtigungen zusammenhängen. Daraus ergab sich die Forschungsfrage: „Welche Faktoren des Verhaltensmodells der Versorgungsinanspruchnahme nach Andersen sind signifikante Prädiktoren für die Intensität der Tagespflegenutzung bei zu Hause lebenden Personen mit leichter kognitiver Beeinträchtigung (MCI), leichter oder moderater Demenz?“

## Methodik

### Design

Die verwendeten Daten stammen aus der Studie Demenz in der Tagespflege bei psychosozialer MAKS-Intervention (DeTaMAKS-Studie), einer deutschlandweiten clusterrandomisierten kontrollierten Studie in Tagespflegeeinrichtungen. Ziel der Studie war der Wirksamkeitsnachweis einer nichtmedikamentösen Gruppentherapie für kognitiv beeinträchtigte Personen (MCI, leichte oder moderate Demenz). Die Stichprobe bestand aus 453 Dyaden von Tagespflegegästen (TP-Gäste) und ihren pflegenden Angehörigen (PA). Alle TP-Gäste besuchten wenigstens für einen Tag/Woche eine TP. Informationen zu Studiendesign und -ablauf können dem Studienprotokoll entnommen werden [2]. Die Ergebnisse der primären Forschungshypothesen wurden bereits veröffentlicht [3, 8]. Die Ethikkommission der Friedrich-Alexander-Universität Erlangen-Nürnberg hat der Durchführung der Studie inkl. datenschutzrechtlicher Aspekte zugestimmt (Votums-Nr.: 170_14B). Die Einverständniserklärungen wurden von allen teilnehmenden TP-Gästen und PA bzw. deren gesetzlicher Vertretung eingeholt. Die erhobenen Daten wurden ausschließlich pseudonymisiert gespeichert und ausgewertet. Die Studie wurde bei ISCRTN registriert (10.1186/ISRCTN16412551).

Die in diesem Beitrag verwendeten Querschnittsdaten stammen vom ersten Erhebungszeitpunkt vor dem Start der Therapie im April 2015.

### Stichprobe

Von den 453 Dyaden, die an der DeTaMAKS-Studie teilnahmen, konnten die Daten von 449 Dyaden für die Hauptanalyse verwendet werden. (Bei 4 Dyaden fehlte die Angabe, wie häufig die TP genutzt wurde). Bestimmte ermöglichende Variablen (Entfernung und Fahrzeit zwischen Wohnung und TP, Regionstyp und Verstädterungsgrad der TP) lagen nur für zusammenlebende Dyaden vor. Daher wurde mit dieser Substichprobe (*n* = 275) eine Sensitivitätsanalyse durchgeführt.

Im Zusatzmaterial online finden sich die Charakteristika der Hauptstichprobe (Tabelle T1) sowie der Substichprobe (Tabelle T2). Die 34 an der Studie teilnehmenden TP wurden aus dem gesamten Bundesgebiet rekrutiert und sind in Tabelle T3 beschrieben.

### Instrumente

Tabelle T4 gibt einen Überblick über die erfassten Variablen und genutzten Instrumente. Für eine genaue Beschreibung der verwendeten Instrumente: [2].

### Statistische Analysen

Die Besuchshäufigkeit der TP in Tagen/Woche stellte die abhängige Variable dar, dichotomisiert (angelehnt an Straubmeier et al. [17]) in Wenignutzung (1 bis 2 Tage/Woche) und Häufignutzung (3 bis 5 Tage/Woche).

Vor der statistischen Analyse wurden einzelne fehlende Werte in den unabhängigen Variablen mittels Regression imputiert. Die auszuwertende Hauptstichprobe umfasste 449 Fälle. Die Substichprobe umfasste 275 Fälle.

Die statistische Analyse erfolgte für die Hauptanalyse sowie für die Sensitivitätsanalyse in den gleichen drei Schritten:

I. Präanalyse: bivariater Vergleich – nichtadjustiert
  Es wurden Gruppenunterschiede zwischen Wenignutzern und Häufignutzern für alle potenziellen Prädiktoren mit dem Chi-Quadrat-Test oder einem t‑Test für unverbundene Stichproben berechnet.
II. Multikollinearitätsprüfung:
  Die unabhängigen Variablen, für die hinsichtlich der Nutzungsintensität entweder signifikante Gruppenunterschiede (*p* < 0,05) oder ein statistischer Trend (*p* < 0,1) bestanden, wurden auf Multikollinearität überprüft. Bei einem signifikanten Korrelationskoeffizienten über 0,5 wurden einzelne Variablen ausgeschlossen [6].
III. Finale Analyse: multivariates Modell – adjustierte Analyse
  Die nach Schritt II verbleibenden Variablen wurden als Prädiktoren in eine binär-logistische Regression mit Nutzungsintensität als abhängiger Variable (kodiert 0: Wenignutzung und 1: Häufignutzung) aufgenommen. Kategoriale Variablen mit mehr als 2 Ausprägungen wurden in eine dichotome Ausprägung umkodiert.

## Ergebnisse

### Hauptanalyse

I. Ergebnisse der Präanalyse
  Im Mittel wurde die TP an 2,29 Tagen besucht (SD: ± 1,29). Damit sind 64,1 % der Dyaden Wenignutzende und 35,9 % Häufignutzende von TP.
  Im Zusatzmaterial online, Tabelle T1, sind signifikante Unterschiede in 11 Variablen sowie 2 trendandeutende Variablen zwischen den beiden Nutzungsgruppen markiert, die in die Multikollinearitätsprüfung eingingen.
II. Ergebnisse der Multikollinearitätsprüfung
  Es zeigte sich, dass das Alter der PA in einem starken Zusammenhang zur Berufstätigkeit der PA (r = −0,648, *p* < 0,001) und dem Verwandtschaftsgrad zum TP-Gast (r = −0,641, *p* < 0,001) stand. Aus Sparsamkeitsgründen wurde entschieden, die Variable Alter der PA beizubehalten.
III. Finale Analyse
  Im finalen Modell resultierte aus der binär-logistischen Regressionsanalyse ein statistisch signifikantes Modell mit 5 signifikanten Prädiktoren (χ^2^ = 74,148 (df: 8); *p* < 0,001). Nagelkerkes Pseudo‑R^2^ lag bei 0,21, d. h., die untersuchten Variablen konnten 21 % der Varianz der Nutzungsintensität erklären (Tab. 1).

**Table 1:** 

	Unstd. B	*p*‑Wert	Odds ratio	KI	Komponente
Familienstand TP-Gast^a^	−0,889**	< 0,001	0,411**	[−0,057–0,879]	Prädisponierend
Bildungsstand (Jahre) PA	0,122**	0,001	1,129**	[1,057–1,202]	Prädisponierend
Pflegestufe^b^	0,987**	< 0,001	2,683**	[2,210–3,156]	Ermöglichend
Dauer der Tagespflegenutzung (Monate)	0,018**	< 0,001	1,018**	[1,008–1,029]	Ermöglichend
NPI	0,087*	0,038	1,091*	[1,009–1,173]	Bedarf
Inanspruchnahme einer Haushaltshilfe	−0,480	0,054	0,619	[0,131–1,107]	Ermöglichend
Inanspruchnahme eines häuslichen Betreuungsdienstes	−0,650	0,077	0,522	[−0,199–1,244]	Ermöglichend
NOSGER	0,043	0,094	1,044	[0,993–1,095]	Bedarf
Konstante	−3,285	< 0,001	0,037	[−1,217–1,212]	–

Durch Selektion ausgeschlossene Variablen: Alter der pA, Inanspruchnahme eines ambulanten Pflegedienstes, Inanspruchnahme einer Angehörigenberatungsstelle. Nagelkerkes Pseudo‑R^2^ = 0,209; 4 Schritte

*Unstd. B* unstandardisierter B‑Koeffizient, *KI* Konfidenzintervall, *TP-Gast* Tagespflegegast, *PA* pflegende Angehörige

*p < 0,05, **p < 0,01

^a^Dichotomisierte Variable Familienstand des TP-Gastes: mit Partner/-in zusammenlebend (verheiratet, in einer Beziehung): 1; ohne Partner/-in lebend (ledig, getrenntlebend, geschieden, verwitwet): 0

^b^Dichotomisierte Variable Pflegestufe: (keine Pflegestufe, Pflegestufe 0, Pflegestufe 1): 0, (Pflegestufe 2, Pflegestufe 3): 1
  Das finale Modell zeigte für folgende Faktoren eine signifikante Assoziation mit der Nutzungsintensität: *Prädisponierende* Faktoren waren der Familienstand der TP-Gäste (lebte der TP-Gast mit Partner/-in zusammen, sank die Wahrscheinlichkeit, die TP häufig zu nutzen) und der Bildungsstand der PA (mit jedem zusätzlichen Jahr mehr Bildung erhöhte sich die Wahrscheinlichkeit der Häufignutzung um ein Achtel). *Ermöglichende* Faktoren waren die Pflegestufe (Wahrscheinlichkeit der Häufignutzung war mehr als verdoppelt bei Pflegestufe 2 oder 3 im Vergleich zu einer niedrigeren oder keiner Pflegestufe) und die bisherige Dauer der Tagespflegenutzung (mit jedem zusätzlichen Monat der Nutzung erhöhte sich die Wahrscheinlichkeit der Häufignutzung um knapp 2 %). *Bedarfsfaktoren* waren psychische und Verhaltenssymptome (bei der Erhöhung des NPI um einen Punkt erhöhte sich die Wahrscheinlichkeit der Häufignutzung um 9 %).

### Sensitivitätsanalyse

In zusammenlebenden Dyaden waren die TP-Gäste im Vergleich zur Hauptstichprobe jünger (*p* = 0,05) und häufiger männlich (*p* = 0,05), ihre PA jedoch im Mittel älter (*p* = 0,001), seltener berufstätig (*p* = 0,05), stärker durch die Pflegesituation belastet (*p* = 0,001) und nahmen seltener ambulante Pflegedienste und Essen auf Rädern in Anspruch (jeweils *p* = 0,05).

I. Ergebnisse der Präanalyse
  Die Substichprobe nutzte die TP im Mittel an 2,22 Tagen (SD: ± 1,22). 65,8 % der Substichprobe gehörten zu den Wenignutzenden und 34,2 % zu den Häufignutzenden.
  Im Zusatzmaterial online, Tabelle T2, sind signifikante Unterschiede in 9 Variablen sowie in 5 trendandeutenden Variablen zwischen den beiden Nutzungsgruppen markiert, die in die Multikollinearitätsprüfung eingingen.
II. Multikollinearitätsprüfung
  Bei der Prüfung auf Multikollinearität zeigte sich, dass 5 unabhängige Variablen stark untereinander korrelieren: Familienstand der TP-Gäste mit Geschlecht der TP-Gäste (r = −0,543, *p* < 0,001), Familienstand der TP-Gäste mit dem Verwandtschaftsgrad (r = 0,600, *p* < 0,001), Alter der PA mit dem Verwandtschaftsgrad (r = −0,689, *p* < 0,001), Alter der PA mit dem Familienstand der TP-Gäste (r = −0,522, *p* < 0,001), Berufstätigkeit der PA mit dem Verwandtschaftsgrad (r = 0,549, *p* < 0,001) und Berufstätigkeit der PA mit dem Alter der PA (r = −0,708, *p* < 0,001). Es wurden die Variablen Geschlecht der TP-Gäste und Alter der PA als ökonomischste Auswahl für die finale Analyse beibehalten.
III. Finale Analyse
  In der finalen Analyse resultierte aus der binär-logistischen Regressionsanalyse ein statistisch signifikantes Modell (χ^2^ = 62,846 (df:7); *p* < 0,001) mit 5 signifikanten Prädiktoren. Nagelkerkes Pseudo‑R^2^ lag bei 0,28. Die in der Sensitivitätsanalyse untersuchten Variablen erhöhen die aufgeklärte Varianz der Nutzungsintensität somit auf 28 % (Tab. 2).

**Table 2:** 

	Unstd. B	*p*‑Wert	Odds ratio	KI	Komponente
Alter des PA	−0,030*	0,020	0,971*	[0,946–0,996]	Prädisponierend
Familienstand des PA^a^	−0,826*	0,022	0,438*	[−0,267–1,142]	Prädisponierend
Pflegestufe^b^	0,945**	0,001	2,573**	[2,001–3,146]	Ermöglichend
Dauer der Tagespflegenutzung (Monate)	0,029**	< 0,001	1,030**	[1,014–1,045]	Ermöglichend
Entfernung (km)^c^	−0,078*	0,010	0,925*	[0,866–0,984]	Ermöglichend
Angehörigenberatungsstelle	−0,923	0,080	0,397	[−0,638–1,432]	Ermöglichend
Haushaltshilfe	−0,666	0,053	0,514	[−0,160–1,188]	Ermöglichend
Konstante	1,459	0,077	4,301	[2,684–5,918]	–

Durch Selektion ausgeschlossene Variablen: Geschlecht des Tagespflegegastes, Dauer der häuslichen Pflege, Inanspruchnahme eines häuslichen Betreuungsdienstes, Inanspruchnahme einer Betreuungsgruppe. Nagelkerkes R^2^ = 0,282; 5 Schritte

*Unstd. B* unstandardisierter B‑Koeffizient, *KI* Konfidenzintervall 95, *PA* pflegende Angehörige

*p < 0,05, **p < 0,01

^a^Dichotomisierte Variable Familienstand des PA: mit Partner/-in zusammenlebend (verheiratet, in einer Beziehung): 1; ohne Partner/-in lebend (ledig, getrenntlebend, geschieden, verwitwet): 0

^b^Dichotomisierte Variable Pflegestufe: (keine Pflegestufe, Pflegestufe 0, Pflegestufe 1): 0, (Pflegestufe 2, Pflegestufe 3): 1

**Figure 2:**
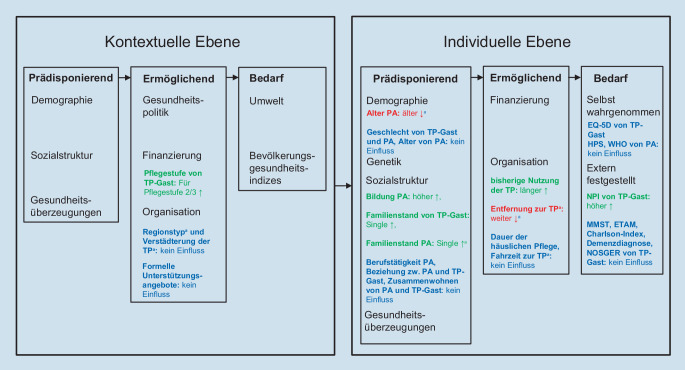

^c^Zwischen Tagespflege und Wohnort
  Das finale Modell zeigte analog zur gesamten Stichprobe den Zusammenhang der Pflegestufe und der bisherigen Dauer der Tagespflegenutzung mit der Nutzungsintensität. Zusätzlich kamen als *prädisponierende* Faktoren das Alter der PA (mit jedem Jahr, das die PA älter waren, verringerte sich die Wahrscheinlichkeit der Häufignutzung), der Familienstand der PA (bei PA, die mit Partner/-in zusammenlebten, sank die Wahrscheinlichkeit der Nutzung) sowie als *ermöglichender* Faktor die Entfernung zwischen Wohnort und TP (mit jedem Kilometer mehr verringerte sich die Wahrscheinlichkeit der Häufignutzung) hinzu.
  Eine Zuordnung der in dieser Studie erhobenen unabhängigen Variablen in das Verhaltensmodell der Versorgungsinanspruchnahme nach Andersen et al. [1] sowie deren Einfluss auf die Intensität der Tagespflegenutzung zeigt Abb. 2.

## Diskussion

Ziel der Analyse war es, Prädiktoren für die Nutzungsintensität von TP bei Personen mit kognitiver Beeinträchtigung und ihren PA zu finden. Dabei stellten sich der Familienstand des TP-Gastes, der Bildungsstand der PA, die Pflegestufe, die bisherige Dauer der Tagespflegenutzung, psychische und Verhaltenssymptome (NPI) sowie bei zusammenwohnenden Dyaden zusätzlich das Alter und der Familienstand der PA und die Entfernung zur TP als signifikante Prädiktoren heraus.

### Prädisponierende Faktoren

Bei Donath et al. und Lüdecke et al. [8, 12] war ein höheres Alter der PA mit der Nutzung von TP assoziiert. Die vorliegende Analyse zeigte bei zusammenlebenden Dyaden jedoch den gegenteiligen Zusammenhang. Dies lässt sich möglicherweise dadurch erklären, dass die jüngeren PA der hier untersuchten Dyaden fast ausschließlich Kinder/Schwiegerkinder, eher berufstätig und somit eher auf eine Betreuung angewiesen waren als die älteren PA, die zumeist Partner/-in des jeweiligen TP-Gastes waren. Dies passt auch zu dem Befund, dass eine TP weniger intensiv genutzt wurde, wenn der TP-Gast in einer Partnerschaft lebt.

Übereinstimmend mit der Literatur [9, 12, 13] zeigen auch die vorliegenden Ergebnisse, dass eine höhere Bildung der PA mit einer höheren Nutzungsintensität korreliert.

### Ermöglichende Faktoren

Der hier gefundene Zusammenhang von erhöhter Nutzung bei höherer Pflegestufe bestätigt sich in der Literatur nicht [4, 12]. Im System der Pflegestufen wurden die Kosten für die TP mit anderen Leistungen der Pflegekasse verrechnet. Diese Verrechnung wurde erst kurz vor Studienbeginn aufgegeben. Eine erhöhte Nutzungsintensität bei Vorliegen höherer Pflegestufen lässt sich somit nur eingeschränkt durch bessere Finanzierungsmöglichkeiten erklären – insbesondere für Dyaden, die die TP schon länger nutzten. Stattdessen wird dies als erhöhter Bedarf interpretiert. Allerdings kann nicht von einem linear steigenden Bedarf ausgegangen werden, da Personen mit höchster Pflegestufe der Besuch einer TP meist nicht mehr möglich ist. Dies zeigt sich auch darin, dass in der hier untersuchten Stichprobe nur 4 Personen die Pflegestufe 3 hatten.

Mit längerer bereits bestehender Nutzung stieg die Intensität der Nutzung der TP. Dies ist ein Hinweis darauf, dass die Nutzung von den PA und/oder den TP-Gästen nach einer Eingewöhnungsphase als positiv empfunden wird. Es scheint eine Anfangshürde zu geben, die zu Beginn eine niedrigere Nutzung mit sich bringt. Derzeit liegen dazu jedoch keine hinreichenden, wissenschaftlichen Erkenntnisse vor. Die beiden ermöglichenden Faktoren Pflegestufe und Nutzungsdauer zeigten sich sowohl in der Haupt- als auch in der Sensitivitätsanalyse als signifikante Prädiktoren, was für eine Belastbarkeit dieser Befunde spricht.

Die Entfernung zur TP als signifikanter ermöglichender Faktor in der Substichprobe deckt sich mit Donath et al. und Phillipson et al. [8, 13] bezüglich der Nutzung/Nichtnutzung, jedoch nicht mit Kremer-Preiß [11]. Sind die PA selbst für die Organisation des Transportes zuständig, werden Fahrten mit größerer Entfernung zeitaufwendiger, schwieriger und im Fall von stark ausgeprägten psychischen und Verhaltenssymptomen bei den TP-Gästen anstrengender. Einige TP bieten einen Transport der TP-Gäste an. Diese Information wurde nicht erhoben, jedoch ist es plausibel, dass der Einfluss der Entfernung zur TP bei einem Transportangebot sinkt.

### Bedarfsfaktoren

Die einzigen signifikanten Bedarfsfaktoren in der Hauptstichprobe waren psychische und Verhaltenssymptome. Bei zusammenwohnenden Dyaden zeigte sich dieser Zusammenhang nicht. Dies ist erstaunlich, da gerade die psychischen und Verhaltenssymptome das Zusammenleben stark belasten können [7]. Die von anderen Autoren [8, 12, 13] identifizierten Zusammenhänge von Tagespflegenutzung und Belastung der PA bzw. den Einschränkungen der TP-Gäste in kognitiven und alltagspraktischen Bereichen konnten in Bezug auf die Nutzungsintensität nicht gefunden werden. Allerdings wurden nur Dyaden in die Studie eingeschlossen, die die TP bereits nutzten. Es ist möglich, dass sich in dieser Studie Bedarfsfaktoren nicht hinreichend identifizieren ließen, da ein Bedarf bei allen Nutzern vorhanden und damit die Varianz diesbezüglich in der Stichprobe gering war. Somit wäre ein Zusammenhang zwischen Nutzungsintensität und Bedarf nicht abbildbar.

## Limitationen

Mit den verwendeten Daten können keine Rückschlüsse auf Nichtnutzende von TP gezogen werden, weil die Stichprobe nur aus Nutzenden von TP bestand. Es handelte sich außerdem um Querschnittsdaten, die keinen kausalen Schluss ermöglichen. Die Daten zu Wohnort sowie räumlicher und zeitlicher Entfernung zur TP konnten nur für einen Teil der Stichprobe erhoben werden. Da der Erhebungszeitpunkt der Daten vor der letzten Reform des Pflegeversicherungsgesetzes lag, waren die Leistungen für die Inanspruchnahme von TP in der vorliegenden Stichprobe deutlich eingeschränkter als gegenwärtig. Zieht man die aktuellen Leistungen für TP in Betracht, ist ein noch stärkerer Einfluss des Pflegegrades auf die Nutzungsintensität der TP anzunehmen, als hier festgestellt wurde. Einige Faktoren des Verhaltensmodells der Versorgungsinanspruchnahme wie Gesundheitspolitik, Umwelt oder Angebotsflexibilität sowie Gesundheitsüberzeugungen oder Genetik konnten auf Basis der verwendeten Daten nicht untersucht werden. Zukünftige Studien könnten diese sowie die Abhängigkeiten zwischen verschiedenen Faktoren in den Blick nehmen.

## Fazit für die Praxis

- Der Bedarf an Betreuung und Förderung durch Tagespflegen ist vorhanden und steigt mit zunehmender Pflegebedürftigkeit.
- Der Bedarf wird in Zukunft durch die verbreitete Berufstätigkeit aller Bevölkerungsgruppen und Lebensformen außerhalb von festen Partnerschaften eher zunehmen.
- Die Entfernung der Tagespflege ist ein signifikanter Prädiktor für die Nutzungsintensität. Um die Nutzung zu erhöhen, müssten Tagespflegen für potenzielle Nutzende schnell erreichbar sein.
- Pflegende Angehörige und Menschen mit kognitiven Beeinträchtigungen sollten die Möglichkeit bekommen, das Angebot zunächst probeweise kennenzulernen. Als Erleichterung des „Einstiegs“ könnten Einrichtungen eine „Testwoche“/einen „Testmonat“ anbieten. Auch flexible Nutzungskonzepte mit z. B. Zeitkontingenten könnten eine nachfolgend stärkere Nutzungsintensität fördern.

## Supplementary Information





## Abkürzungen

MCI: „Mild cognitive impairment“ (leichte kognitive Beeinträchtigung)
PA: Pflegende Angehörige
PmD: Person mit Demenz
TP: Tagespflege
TP-Gäste: Tagespflegegäste
