# Supplementary material for: Predictors of intensity of use of adult day care centers in people with cognitive impairment
Source: Z Gerontol Geriatr. 2021 Sep 29;55(7):575–82. [Article in German] doi: 10.1007/s00391-021-01972-x (PMC9587102; doi:10.1007/s00391-021-01972-x)
Supplement: Supplementary file 1 [file 391_2021_1972_MOESM1_ESM.docx]

# Appendix: Tabellen zusätzlich

Tabelle T1: Deskriptive Statistik der Hauptstichprobe (n=449 Dyaden, 34 TP)

| Charakteristik | Wenig Nutzung der Tagespflege (1-2 Tage/Woche)  (n=288, 64,1%) | Häufig Nutzung der Tagespflege (3-5Tage/Woche)  (n=161, 35,9%) | Gruppenunterschiede  p-Wert | Gesamte Stichprobe  (n=449) |
| --- | --- | --- | --- | --- |
| Tagespflegegäste |  |  |  |  |
| Alter, M (SD) | 81,8 (7,0) | 81,5 (8,8) | 0,729^a^ | 81,7 (7,7) |
| weiblich^d^, N (%) | 172 (59,7) | 104 (64,6) | 0,309^b^ | 276 (61,5) |
| Bildungsstand (Jahre), M (SD) | 9,8 (2,5) | 9,7 (2,2) | 0,470^a^ | 9,8 (2,4) |
| Beziehung: Angehörige/r ist…, N (%) |  |  | 0,026*^b^ |  |
| Ehepartner/in | 89 (30,9) | 31 (19,3) |  | 120 (26,7) |
| Kind/ Schwiegerkind | 183 (63,5) | 118 (73,3) |  | 301 (67,0) |
| Sonstige^c^ | 16 (5,6) | 12 (7,5) |  | 28 (6,2) |
| Demenzdiagnose bekannt, N (%) | 178 (61,8) | 109 (67,7) | 0,212^b^ | 287 (63,9) |
| Familienstand, N (%) |  |  | 0,009**^b^ |  |
| ledig | 8 (2,8) | 7 (4,3) |  | 15 (3,3) |
| in Beziehung/ verheiratet | 132 (45,8) | 48 (29,8) |  | 180 (40,1) |
| getrennt lebend/ geschieden | 6 (2,1) | 6 (3,7) |  | 12 (2,7) |
| verwitwet | 142 (49,3) | 100 (62,1) |  | 242 (53,9) |
| Dauer der häuslichen Pflege (Monate), M (SD) | 56,1 (49,0) | 64,2 (52,6) | 0,103^a^ | 59,0 (50,4) |
| Pflegende Angehörige |  |  |  |  |
| Alter, M (SD) | 60,6 (12,1) | 57,7 (10,2) | 0,006**^a^ | 59,6 (11,5) |
| weiblich^d^, N (%) | 216 (75,0) | 112 (69,6) | 0,213^b^ | 328 (73,1) |
| berufstätig, N (%) | 144 (50,0) | 99 (61,5) | 0,019*^b^ | 243 (54,1) |
| Familienstand, N (%) |  |  | 0,172^b^ |  |
| ledig | 20 (6,9) | 16 (9,9) |  | 36 (8,0) |
| in Beziehung/ verheiratet | 236 (81,9) | 120 (74,5) |  | 356 (79,3) |
| getrennt lebend / geschieden | 21 (7,3) | 20 (12,4) |  | 41 (9,1) |
| verwitwet | 11 (3,8) | 5 (3,1) |  | 16 (3,6) |
| Wohnsituation: Zusammenwohnend, N (%) | 181 (62,8) | 94 (58,4) | 0,352^b^ | 275 (61,2) |
| Bildungsstand (Jahre), M (SD) | 10,7 (2,6) | 11,3 (3,3) | 0,026*^a^ | 10,9 (2,9) |
| Ermöglichende Faktoren |  |  |  |  |
| Pflegestufe, N (%) |  |  | 0,002**^b^ |  |
| keine Pflegestufe | 19 (6,6) | 4 (2,5) |  | 23 (5,1) |
| Pflegestufe 0 | 35 (12,2) | 13 (8,1) |  | 48 (10,7) |
| Pflegestufe 1 | 162 (56,2) | 75 (46,6) |  | 237 (52,8) |
| Pflegestufe 2 | 70 (24,3) | 67 (41,6) |  | 137 (30,5) |
| Pflegestufe 3 | 2 (0,7) | 2 (1,2) |  | 4 (0,9) |
| Dauer der Tagespflegenutzung (Monate), M (SD) | 18,7 (18,8) | 29,4 (28,1) | <0,001**^a^ | 22,6 (23,1) |
| Summe genutzte Unterstützungsangebote, M (SD) | 1,7 (1,3) | 1,5 (1,2) | 0,085^a^ | 1,6 (1,3) |
| Inanspruchnahme Angehörigenberatungsstelle, N (%) | 50 (17,4) | 13 (8,1) | 0,007**^b^ | 63 (14,0) |
| Inanspruchnahme Haushaltshilfe, N (%) | 96 (33,3) | 36 (22,4) | 0,014*^b^ | 132 (29,4) |
| Inanspruchnahme häuslicher Betreuungsdienst, N (%) | 44 (15,3) | 12 (7,5) | 0,016*^b^ | 56 (12,5) |
| Inanspruchnahme ambulanter Pflegedienst, N (%) | 128 (44,4) | 85 (52,8) | 0,089+^b^ | 213 (47,4) |
| Inanspruchnahme Betreuungsgruppe, N (%) | 26 (9,0) | 8 (5,0) | 0,119^b^ | 34 (7,6) |
| Inanspruchnahme Kurzzeitpflege, N (%) | 45 (15,6) | 32 (19,9) | 0,252^b^ | 77 (17,1) |
| Teilnahme Pflegekurs, N (%) | 39 (13,5) | 17 (10,6) | 0,359^b^ | 56 (12,5) |
| Inanspruchnahme Angehörigengruppe, N (%) | 19 (6,6) | 13 (8,1) | 0,560^b^ | 32 (7,1) |
| Inanspruchnahme Essen auf Rädern, N (%) | 42 (14,6) | 22 (13,7) | 0,789^b^ | 64 (14,3) |
| Bedarfsfaktoren |  |  |  |  |
| NOSGER, M (SD) | 15,3 (4,2) | 16,2 (4,6) | 0,038*^a^ | 15,6 (4,4) |
| NPI, M (SD) | 5,2 (2,7) | 5,7 (2,8) | 0,051+^a^ | 5,4 (2,7) |
| Charlson-Index, M (SD) | 2,2 (1,6) | 2,4 (1,6) | 0,296^a^ | 2,3 (1,6) |
| ETAM, M (SD) | 17,6 (7,1) | 16,8 (7,3) | 0,283^a^ | 17,3 (7,2) |
| MMST, M (SD) | 19,6 (4,7) | 19,2 (4,7) | 0,391^a^ | 19,4 (4,7) |
| WHO-5, M (SD) | 11,6 (5,9) | 11,9 (6,3) | 0,623^a^ | 11,7 (6,0) |
| EQ-5D Tagespflegebesucher, M (SD) | 0,4 (0,2) | 0,4 (0,2) | 0,704^a^ | 0,4 (0,2) |
| HPS, M (SD) | 12,6 (8,3) | 12,6 (7,7) | 0,950^a^ | 12,6 (8,1) |

*Anmerkungen.* M = Mittelwert, SD = Standardabweichung, + = p<0,1, * = p<0,05, ** = p<0,01.

^a^ T-Test

^b^ Chi-Quadrat-Test

^c^ beinhaltet andere Verwandte und Nicht-Verwandte (befreundete Personen, Bekannte)

^d^ Geschlecht binär erfasst

MMST = Mini Mental Status Test, ETAM = Erlangen Test of Activities of Daily Living in Persons with Mild Dementia or Mild Cognitive Impairment, NOSGER = Nursing Observation Scale for Geriatric Persons, NPI = Neuropsychiatrisches Inventar, HPS = Häusliche Pflegeskala, WHO-5 = WHO Depressionsskala, Charlson-Index = Charlson Komorbiditätsindex, EQ-5D = Gesundheitsfragebogen der EuroQoL Group.

Tabelle T2: Deskriptive Statistik der Substichprobe zusammenwohnender Dyaden (n=275 Dyaden, 34 TP)

| Charakteristik | Wenig Nutzung der Tagespflege (1-2 Tage/Woche)  (n=181, 65,8%) | Häufig Nutzung der Tagespflege (3-5Tage/Woche)  (n=94, 34,2%) | Gruppenunterschiede  p- Wert | Zusammenwohnende Dyaden  (n=275) |
| --- | --- | --- | --- | --- |
| Tagespflegegäste |  |  |  |  |
| Alter (Jahre), M (SD) | 80,7 (7,5) | 79,8 (9,6) | 0,449^a^ | 80,4 (8,2) |
| weiblich^d^, N (%) | 87 (48,1) | 57 (60,6) | 0,048*^b^ | 144 (52,4) |
| Familienstand, N (%) |  |  | 0,098+^b^ |  |
| Ledig | 5 (2,8) | 3 (3,2) |  | 8 (2,9) |
| in Beziehung/ verheiratet | 101 (55,8) | 40 (42,6) |  | 141 (51,3) |
| getrennt lebend/ geschieden | 2 (1,1) | 4 (4,3) |  | 6 (2,2) |
| Verwitwet | 73 (40,3) | 47 (50,0) |  | 120 (43,6) |
| Demenzdiagnose bekannt, N (%) | 118 (65,2) | 63 (67,0) | 0,762^b^ | 181 (65,8) |
| Beziehung: Angehörige/r ist…, N (%) |  |  | 0,035*^b^ |  |
| Ehepartner/in | 89 (49,2) | 31 (33,0) |  | 120 (43,6) |
| Kind/ Schwiegerkind | 85 (47,0) | 59 (62,8) |  | 144 (52,4) |
| Sonstige | 7 (3,9) | 4 (4,3) |  | 11 (4,0) |
| Bildungsstand (Jahre), M (SD) | 10,1 (2,8) | 9,7 (2,3) | 0,163^a^ | 10,0 (2,6) |
| Dauer häusliche Pflege (Monate), M (SD) | 57,3 (52,5) | 69,9 (52,4) | 0,060+^a^ | 61,6 (52,7) |
| Pflegende Angehörige |  |  |  |  |
| Alter (Jahre), M (SD) | 64,2 (12,4) | 59, 2 (11,8) | 0,002**^a^ | 62,5 (12,4) |
| weiblich^d^, N (%) | 137 (75,7) | 67 (71,3) | 0,428^b^ | 204 (74,2) |
| Familienstand, N (%) |  |  | 0,039*^b^ |  |
| Ledig | 9 (5,0) | 13 (13,8) |  | 22 (8,0) |
| in Beziehung/ verheiratet | 154 (85,1) | 68 (72,3) |  | 222 (80,7) |
| getrennt lebend/ geschieden | 11 (6,1) | 9 (9,6) |  | 20 (7,3) |
| verwitwet | 7 (3,9) | 4 (4,3) |  | 11 (4,0) |
| Berufstätig, N (%) | 69 (38,1) | 53 (56,4) | 0,004**^b^ | 122 (44,4) |
| Bildungsstand (Jahre), M (SD) | 10,6 (2,7) | 10,9 (3,1) | 0,425^a^ | 10,7 (2,9) |
| Ermöglichende Faktoren |  |  |  |  |
| Pflegestufe, N (%) |  |  | 0,001**^b^ |  |
| keine Pflegestufe | 13 (7,2) | 1 (1,1) |  | 14 (5,1) |
| Pflegestufe 0 | 20 (11,0) | 7 (7,4) |  | 27 (9,8) |
| Pflegestufe 1 | 92 (50,8) | 34 (36,2) |  | 126 (45,8) |
| Pflegestufe 2 | 54 (29,8) | 50 (53,2) |  | 104 (37,8) |
| Pflegestufe 3 | 2 (1,1) | 2 (2,1) |  | 4 (1,5) |
| Dauer der Tagespflegenutzung (Monate), M (SD) | 18,6 (17,5) | 31,7 (26,5) | <0,001**^a^ | 23,1 (21,8) |
| Summe angenommener Unterstützungsangebote, M (SD) | 1,7 (1,4) | 1,4 (1,2) | 0,073^a^ | 1,6 (1,4) |
| Teilnahme Pflegekurs, N (%) | 31 (17,1) | 11 (11,7) | 0,236^b^ | 42 (15,3) |
| Inanspruchnahme Angehörigenberatungsstelle, N (%) | 34 (18,8) | 5 (5,3) | 0,002**^b^ | 39 (14,2) |
| Inanspruchnahme Angehörigengruppe, N (%) | 16 (8,8) | 10 (10,6) | 0,629^b^ | 26 (9,5) |
| Inanspruchnahme häuslicher Betreuungsdienst, N (%) | 33 (18,2) | 8 (8,5) | 0,032*^b^ | 41 (14,9) |
| Inanspruchnahme Betreuungsgruppe, N (%) | 18 (9,9) | 4 (4,3) | 0,099+^b^ | 22 (8,0) |
| Inanspruchnahme Essen auf Rädern, N (%) | 15 (8,3) | 8 (8,5) | 0,949^b^ | 23 (8,4) |
| Inanspruchnahme Kurzzeitpflege, N (%) | 36 (19,9) | 25 (26,6) | 0,204^b^ | 61 (22,2) |
| Inanspruchnahme ambulanter Pflegedienst, N (%) | 64 (35,4) | 40 (42,6) | 0,243^b^ | 104 (37,8) |
| Inanspruchnahme Haushaltshilfe, N (%) | 54 (29,8) | 18 (19,1) | 0,056+^b^ | 72 (26,2) |
| Verstädterungsgrad^e^, N (%) |  |  | 0,129^b^ |  |
| gering besiedelt | 24 (13,3) | 7 (7,4) |  | 31 (11,3) |
| mittlere Besiedlungsdichte | 76 (42,0) | 34 (36,2) |  | 110 (40,0) |
| dicht besiedelt | 81 (44,8) | 53 (56,4) |  | 134 (48,7) |
| Entfernung (km)^f^, M (SD) | 6,9 (5,3) | 5,8 (4,9) | 0,093+^a^ | 6,5 (5,2) |
| Region^e^, M (SD) |  |  | 0,439^b^ |  |
| Landgemeinde | 22 (12,2) | 6 (6,4) |  | 28 (10,2) |
| Kleinstadt | 47 (26,0) | 23 (24,5) |  | 70 (25,5) |
| Mittelstadt | 46 (25,4) | 25 (26,6) |  | 71 (25,8) |
| Großstadt | 66 (36,5) | 40 (42,6) |  | 106 (38,5) |
| Fahrzeit ^f g^, M (SD) | 1,9 (1,4) | 1,8 (1,6) | 0,424^a^ | 1,9 (1,5) |
| Bedarfsfaktoren |  |  |  |  |
| MMST, M (SD) | 19,2 (4,5) | 18,5 (4,5) | 0,173^a^ | 19,0 (4,5) |
| ETAM, M (SD) | 16,8 (7,0) | 15,6 (7,1) | 0,179^a^ | 16,4 (7,1) |
| NOSGER, M (SD) | 15,8 (4,3) | 16,5 (4,6) | 0,205^a^ | 16,1 (4,4) |
| NPI, M (SD) | 5,5 (2,6) | 5,8 (2,9) | 0,404^a^ | 5,6 (2,8) |
| HPS, M (SD) | 14,5 (7,8) | 14,0 (7,0) | 0,564^a^ | 14,3 (7,5) |
| WHO-5, M (SD) | 10,8 (5,6) | 11,1 (5,7) | 0,658^a^ | 10,9 (5,6) |
| Charlson-Index, M (SD) | 2,3 (1,6) | 2,3 (1,5) | 0,846^a^ | 2,3 (1,5) |
| EQ-5D Tagespflegebesucher, M (SD) | 0,4 (0,2) | 0,4 (0,2) | 0,692^a^ | 0,4 (0,2) |

km Kilometer, M Mittelwert, SD Standardabweichung, + = p<0,1, * = p<0,05, ** = p<0,01.

^a^ T-Test

^b^ Chi-Quadrat-Test

^c^ beinhaltet andere Verwandte und Nicht-Verwandte (befreundete Personen, Bekannte)

^d^ Geschlecht binär erfasst

^e^ der Tagespflege

^f^ zwischen Tagespflege und Wohnort

^g^ Einteilung in 5-Minuten-Intervalle (von ≤ 5 Min = 0, bis > 60 Min =12)

MMST Mini Mental Status Test, ETAM Erlangen Test of Activities of Daily Living in Persons with Mild Dementia or Mild Cognitive Impairment, NOSGER Nursing Observation Scale for Geriatric Persons, NPI Neuropsychiatrisches Inventar, HPS Häusliche Pflegeskala, WHO-5 WHO Depressionsskala, Charlson-Index Charlson Komorbiditätsindex, EQ-5D Gesundheitsfragebogen der EuroQoL Group

Tabelle T3: Deskriptive Statistik der Tagespflegen (n =34)

| Charakteristik | Minimum | Maximum | Median | M (SD) |
| --- | --- | --- | --- | --- |
| Plätze vorhanden | 12 | 34 | 15,5 | 18,0 (6,2) |
| Durchschnittliche Anzahl besetzter Plätze pro Tag, N | 8 | 34 | 15 | 16,3 (6,1) |
| Durchschnittliche Auslastung pro Tag, % | 45 | 107,7 | 90,3 | 89,2 (13,8) |
| Anzahl rekrutierter Dyaden, N | 3 | 28 | 13 | 13,3 (5,9) |
| Anzahl der Verträge nach Tagen |  |  |  |  |
| 1 Tag | 0 | 60 | 10,5 | 12,7 (11,3) |
| 2 Tage | 2 | 26 | 11 | 12,1 (6,5) |
| 3 Tage | 2 | 16 | 7 | 6,9 (2,7) |
| 4 Tage | 0 | 9 | 2 | 2,4 (2,2) |
| 5 Tage | 0 | 17 | 4 | 4,1 (3,1) |
| Personen auf der Warteliste | 0 | 40 | 2 | 3,3 (7,1) |

*Anmerkungen.* M = Mittelwert, SD = Standardabweichung.

Tabelle T4: Verfügbare unabhängige Variablen

| Kategorie | Verfügbare unabhängige Variablen für TP-Gast | Verfügbare unabhängige Variablen für PA |
| --- | --- | --- |
| Soziodemographische Daten | Alter in Jahren | Alter in Jahren |
|  | Familienstand | Familienstand |
|  | Bildungsstand in Jahren | Bildungsstand in Jahren |
|  | Geschlecht | Geschlecht |
|  |  | Berufstätigkeit |
|  |  | Verwandtschaftsgrad zum TP-Gast |
|  |  | Zusammenwohnen mit TP-Gast |
| Spezifika der Pflegesituation | Dauer der häuslichen Pflege in Monaten |  |
|  | Bisherige Nutzung der Tagespflege in Monaten |  |
|  | Pflegestufe |  |
|  | Nutzung verschiedener Unterstützungsangebote (FIMA) |  |
| Gesundheitliche Situation | Vorliegen einer Demenzdiagnose | Subjektive Belastung (HPS) |
|  | Komorbiditäten (Charlson-Index) |  |
|  | Kognitiver Status (MMST) |  |
|  | Alltagspraktische Fähigkeiten (ETAM) |  |
|  | Sozialverhalten (NOSGER) |  |
|  | psychische und Verhaltenssymptome (NPI) |  |
|  | Depressivität (WHO-5) |  |
|  | gesundheitsbezogene Lebensqualität (EQ-5D-5L) |  |
| Substichprobe | Entfernung zwischen Wohnort und Tagespflege in km | |
|  | Fahrzeit zwischen Wohnort und Tagespflege in Fünf-Minuten-Intervallen | |
|  | Region der besuchten Tagespflege | |
|  | Verstädterungsgrad der besuchten Tagespflege | |

*Anmerkungen.* TP-Gast: Tagespflege-Gast, PA: pflegende Angehörige

MMST = Mini Mental Status Test, ETAM = Erlangen Test of Activities of Daily Living in Persons with Mild Dementia or Mild Cognitive Impairment, NOSGER = Nursing Observation Scale for Geriatric Persons, NPI = Neuropsychiatrisches Inventar, HPS = Häusliche Pflegeskala, WHO-5 = WHO Depressionsskala, Charlson-Index = Charlson Komorbiditätsindex, EQ-5D = Gesundheitsfragebogen der EuroQoL Group.
